# Prevalence and characteristics of surgical site hypervirulent *Klebsiella pneumoniae* isolates

**DOI:** 10.1002/jcla.23364

**Published:** 2020-05-19

**Authors:** Qiang Zhao, Ling Guo, Li‐Feng Wang, Qian Zhao, Ding‐Xia Shen

**Affiliations:** ^1^ Chinese PLA General Hospital Beijing China

**Keywords:** hypervirulent, *Klebsiella pneumoniae*, surgical site infection

## Abstract

**Background:**

We aim to determine the prevalence of hypervirulent *Klebsiella pneumoniae* (hvKp), which causes surgical site infections (SSIs), and describe the microbiological and molecular characteristics of hvKp isolates.

**Methods:**

Non‐duplicate *K. pneumoniae* strains were isolated from wound drainage specimens of postoperative patients at the Chinese PLA General Hospital between September 2008 and July 2017. Antimicrobial susceptibility, string test, pulsed‐field gel electrophoresis (PFGE), and genome sequencing analyses were performed.

**Results:**

Fifty‐one *K. pneumoniae* strains were isolated from wound drainage specimens collected from postoperative patients. Twenty‐six hvKp strains, including 17 (17/37, 46.0%) and 9 (9/14, 64.3%) hvKp strains, were isolated from 37 and 14 patients with SSIs and community‐acquired infections (CAIs), respectively. Notably, 4 extended‐spectrum beta‐lactamase (ESBL)‐producing hvKp strains (4/26, 15.4%) and 2 carbapenem‐resistant hvKp strains (2/26, 7.7%) were found. Thirteen K1 serotype (13/26, 50.0%) and 7 K2 serotype (7/26, 26.9%) strains were identified. Phylogenetic analysis results showed that 13 K1 serotype isolates exhibited a high degree of clonality, while 7 K2 serotype strains were genetically unrelated. MLST analysis indicated that there was a strong correlation between ST23 and the K1 serotype. ST65, ST86, and ST375 were prevalent in K2 serotype strains. Almost all hvKp strains (24/26, 92.3%) harbored large virulence plasmids with a high degree of homology to pNTUH‐K2044 and sizes ranging from 140 to 220 kbp.

**Conclusions:**

HvKp strains were prevalent in SSIs. Effective surveillance and control measures should be implemented to prevent the dissemination of such organisms, including the ESBL‐producing and carbapenem‐resistant hvKp strains.

## 1 INTRODUCTION

The new variant of *Klebsiella pneumoniae*, hypervirulent *K. pneumoniae* (hvKp), is characterized by its ability to cause life‐threatening, metastatic, and invasive infections, such as liver abscesses, pneumonia, meningitis, necrotizing fasciitis, and endophthalmitis in healthy individuals in the community.^1^ After it was first described in Taiwan in 1986,^2^ the hvKp epidemic has spread in Asia, and has reportedly spread sporadically and at increasing rates in many other countries in North America, Europe, South America, Middle East, and Australia. The hvKp strain is different from classic *K. pneumoniae* (cKp), as it typically exhibits a hypermucoviscosity phenotype, generally identified using the “string test.” Initially, hvKp has been defined by its hypermucoviscosity phenotype^3^, ^4^; however, it is unclear whether all hvKp strains exhibit hypermucoviscosity.^1^ Several putative virulence genes, including the regulator genes of the mucoid phenotype A (rmpA) and siderophore aerobactin (iucABCD), have been found to contribute to hvKp virulence^5^, ^6^ and are located in a large virulent plasmid. Meanwhile, aerobactin, which accounts for >90% of siderophore production,^7^ significantly enhances the ex vivo survival of hvKp in human ascites and serum, and enhances in vivo survival in mice models of systemic and pulmonary infection.^8^ This suggests that aerobactin could be regarded as a crucial virulence factor and new defining characteristic for hvKp.^9^, ^10^ Here, hvKp was defined based on the presence of at least two of the following indicators: a positive string test, rmpA/rmpA2 gene‐positive status, and/or aerobactin‐positive status.

In contrast to cKp, hvKp is rarely resistant to commonly used antimicrobial agents, with the exception of ampicillin. However, extended‐spectrum beta‐lactamase (ESBL)‐producing and carbapenem‐resistant hvKp strains have been observed, especially with healthcare‐associated infections (HAIs), in long‐term care facilities.^11^, ^12^, ^13^ Surgical site infections (SSIs), one of the most frequent types of HAIs, greatly increase the burden on the patient and healthcare system, while the prevalence and molecular characteristics of SSI‐causing hvKp isolates remain limited. This study aims to investigate the prevalence of hvKp‐causing SSIs and describe the microbiological and molecular characteristics of hvKp isolates.

## 2 MATERIALS AND METHODS

### 2.1 Bacterial isolates and antimicrobial susceptibility testing

Fifty‐one non‐duplicate *K. pneumoniae* strains were isolated from wound drainage specimens of postoperative patients at the Chinese PLA General Hospital, between September 2008 and July 2017. Bacterial identification was performed using MALDI‐TOF (bioMérieux), and results were further confirmed by 16S rRNA gene sequencing. The ESBL production level and antimicrobial susceptibility to ceftazidime, ceftriaxone, cefepime, aztreonam, ertapenem, imipenem, amikacin, gentamicin, ciprofloxacin, levofloxacin, and sulfamethoxazole‐trimethoprim were assessed using Vitek II (bioMérieux), and results were interpreted according to the 2018 Clinical and Laboratory Standards Institute (CLSI) guidelines.

### 2.2 Case definitions and clinical data collection

According to the US Centers for Disease Control and Prevention (CDC), SSI is an infection that occurs within 30 days after the operative procedure and involves at least one of the following: purulent drainage; organisms isolated in wound culture; at least one of the following signs of infection: erythema, heat, pain, and swelling of surgical incision; or diagnosis of a SSI by the surgeon.^14^ An abscess observed in a surgical site before the operation should be considered a CAI instead of an SSI. For each case, the following data elements were collected: (a) basic demographic characteristics (age and gender); (b) underlying diseases; (c) surgical site; and (d) hospital outcomes.

### 2.3 String test

The string test was used to identify the hypermucoviscosity phenotype. Positive results were obtained if a viscous string with a length >5mm was formed, by the stretching of bacterial colonies on a blood agar plate after overnight incubation, using an inoculation loop.

### 2.4 Pulsed‐field gel electrophoresis (PFGE)

For all hvKp isolates, PFGE was performed to analyze the genetic relatedness, and S1‐PFGE was used to determine the number and size of plasmids carried by these isolates, as described previously.^15^, ^16^ The PFGE patterns were analyzed using BioNumerics software v7.6 (Applied Maths), and isolates sharing more than 80% similarity were defined to have the same PFGE pattern.

### 2.5 Whole genomic sequencing and analysis

Genomic DNA was extracted using the TIANamp Bacteria DNA Kit (Tiangen Biotech (Beijing) Co., Ltd.), according to the manufacturer's instructions; fragmented via ultrasonication; and sequenced using the Illumina HiSeqXTen system, using a 2 × 150‐bp paired‐end approach. The sequencing reads were assembled de novo using SPAdes 3.12, and subsequently annotated with Prokka 1.12. Resistance genes and plasmid incompatibility groups were predicted using ResFinder 2.1 and PlasmidFinder 1.2 softwares at the Centre for Genomic Epidemiology. The capsular type, multilocus sequence type (MLST), virulence genes, and Yersiniabactin ICEKp structures were predicted using Kleborate (https://github.com/katholt/Kleborate). BLASTN atlases of the pNTUH‐K2044 virulence plasmid were constructed using BLAST Ring Image Generator (BRIG) v0.95 and manually inspected with mauve.

## 3 RESULTS

### 3.1 Clinical and microbiological characteristics of hvKp isolates

Fifty‐one *K. pneumoniae* strains were isolated from wound drainage specimens of postoperative patients between September 2008 and July 2017. These included 37 SSI‐causing and 14 CAI‐causing strains. Among these, 26 strains (26/51, 51.0%) were identified as hvKp strains. Moreover, 17 (17/37, 46.0%) and 9 (9/14, 64.3%) hvKp strains were isolated from 37 SSI and 14 CAI patients, respectively. Demographic data and clinical characteristics have been shown in Table 1. Thirty‐six (70.6%) patients were males, and the mean age was 49.5 ± 15.6 years. Thirty‐eight patients (74.5%) had cancer, while 12 (23.5%) had diabetes. Ten patients (19.6%) had no underlying diseases. The major surgical sites included the large bowel (29.4%), head and neck (23.5%), and abdominal cavity (17.6%). There were no significant differences in the secondary surgery incidence, hospitalization duration, and 30‐day mortality values between patients infected with hvKp and cKp (Table 1).

**TABLE 1:** Demographic data and clinical characteristics of patients infected with hvKp vs cKp

Characteristics	No. of patients (n, %)			*P* value
	Total (n = 51)	With hvKp infection (n = 26)	With cKp infection (n = 25)	
Basic demographics				
Age (y, mean ± SD)	49.5 ± 15.6	46.0 ± 16.7	53.2 ± 13.8	.105
Male	36 (70.6)	18 (69.2)	18 (72.0)	.828
Underlying diseases				
Diabetes	12 (23.5)	9 (34.6)	3 (12.0)	.057
Cardiovascular disease	10 (19.6)	4 (15.4)	6 (24.0)	.673
Cancer	38 (74.5)	18 (69.2)	20 (80.0)	.378
None	10 (19.6)	6 (23.1)	4 (16.0)	.777
Surgical site				
Large bowel	15 (29.4)	6 (23.1)	9 (36.0)	
Head and neck	12 (23.5)	7 (26.9)	5 (20.0)	
Abdominal cavity	9 (17.6)	5 (19.2)	4 (16.0)	
Orthopedics	5 (9.8)	3 (11.5)	2 (8.0)	
Oral cavity	4 (7.8)	2 (7.7)	2 (8.0)	
Uterus and adnexa	3 (5.9)	1 (3.8)	2 (8.0)	
Gastric and small bowel	3 (5.9)	2 (7.7)	1 (4.0)	
Admitted into the critical care unit	10 (19.6)	5 (19.2)	5 (20.0)	.945
Secondary surgery	8 (15.7)	2 (7.7)	6 (24.0)	.224
Duration of hospitalization (day, median (range))	32.0 (6‐231)	23.5 (7‐231)	35.0 (6‐152)	.254
30‐day mortality	0 (0.0)	0 (0.0)	0 (0.0)	

The antimicrobial resistance rates of ceftazidime, ceftriaxone, aztreonam, and gentamicin in patients infected with cKp were significantly higher than those for patients infected with hvKp strains (*P* < .05), while there were no significant differences in antimicrobial resistance rates of amikacin, cefepime, ertapenem, imipenem, ciprofloxacin, levofloxacin, and sulfamethoxazole/trimethoprim for patients infected with hvKp and cKp (Table 2). Notably, 4 ESBL‐producing hvKp strains (4/26, 15.4%) and 2 carbapenem‐resistant hvKp strains (2/26, 7.7%) were detected.

**TABLE 2:** Antimicrobial resistance profiles of 26 hvKp and 25 cKp isolates

Antimicrobial agents	No. of resistant isolates (n, %)		*P* value
	hvKp (n = 26)	cKp (n = 25)	
Ceftazidime	3 (11.5)	9 (36.0)	.040
Ceftriaxone	6 (23.1)	13 (52.0)	.033
Cefepime	2 (7.7)	5 (20.0)	.384
Aztreonam	3 (11.5)	10 (40.0)	.020
Ertapenem	2 (7.7)	3 (12.0)	.963
Imipenem	2 (7.7)	3 (12.0)	.963
Amikacin	2 (7.7)	4 (16.0)	.627
Gentamicin	3 (11.5)	9 (36.0)	.040
Ciprofloxacin	3 (11.5)	8 (32.0)	.076
Levofloxacin	2 (7.7)	5 (20.0)	.384
Sulfamethoxazole/trimethoprim	4 (15.4)	9 (36.0)	.091

### 3.2 Phylogenetic characteristics of hvKp isolates

**FIGURE 1:**
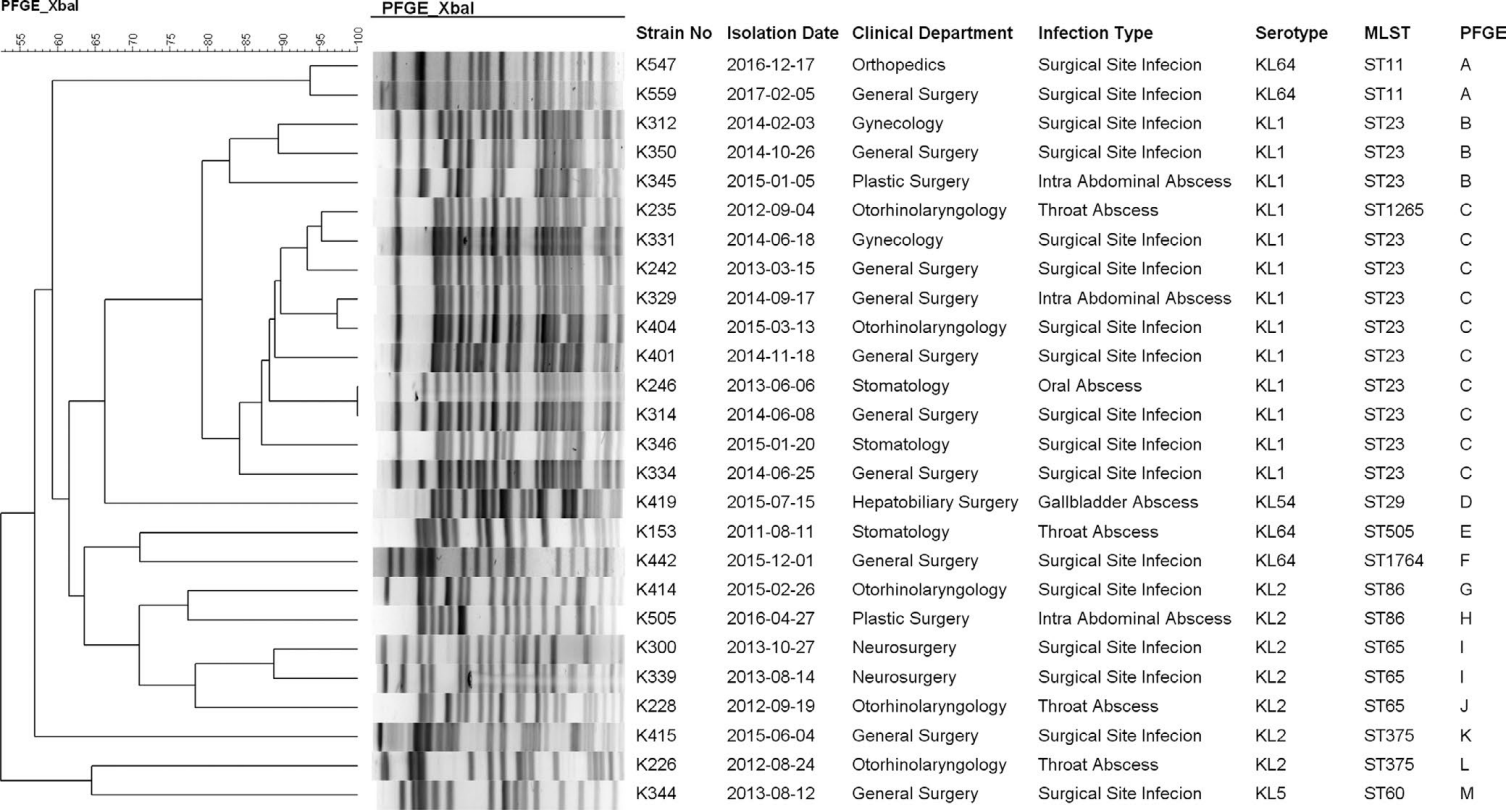
PFGE results of hvKp isolates

Among 26 hvKp isolates, 13, 7, 1, 1, and 4, K1 serotype, K2 serotype, K5 serotype, K54 serotype, and K64 serotype strains were identified, respectively. Phylogenetic analysis showed that 26 hvKp strains revealed 13 different patterns. Thirteen K1 isolates exhibited 2 PFGE patterns, in which 10 strains showed a single pattern. Moreover, all the K1 serotype isolates were found to cluster in a single clade and were clearly separated from the other isolates. This revealed the high degree of clonality of the K1 serotype; the 7 K2 serotype strains were found to be genetically unrelated (Figure 1). MLST analysis identified 10 STs among the 26 hvKp isolates. The most prevalent ST in this study was ST23 (n = 12; 46.2%), followed by ST65 (n = 3; 11.5%), ST375 (n = 2; 7.7%), ST86 (n = 2; 7.7%), and ST11 (n = 2; 7.7%). There was a strong correlation between ST23 and the K1 serotype. ST1265, which shared 6 alleles with ST23, was also associated with the K1 serotype. ST65, ST86, and ST375 were prevalent STs in K2 serotype strains.

### 3.3 Virulence characteristics of hvKp isolates

**FIGURE 2:**
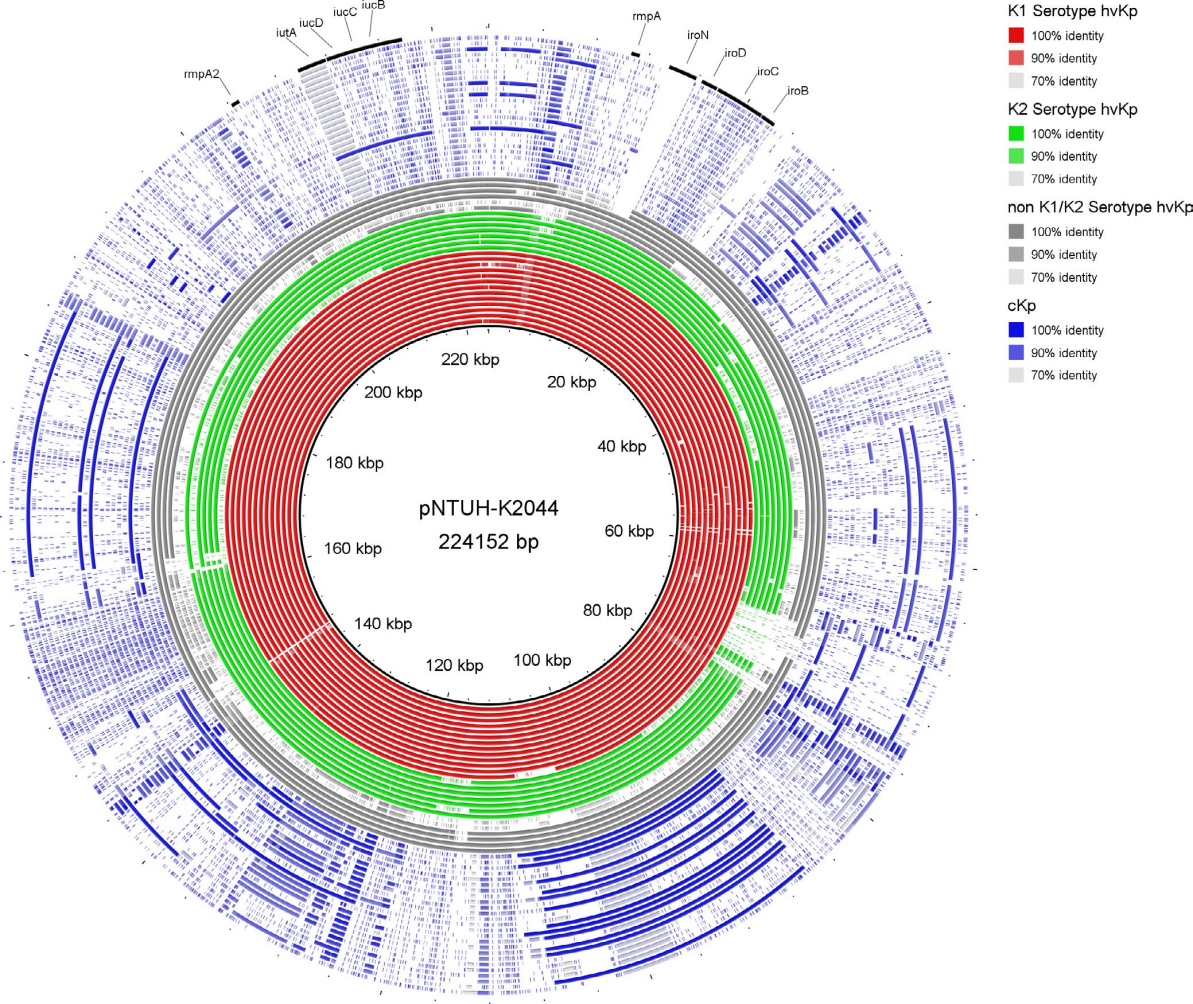
BLASTN atlas illustrating the presence of the pNTUH‐K2044 virulence plasmid in hvKp (K1 serotype [red rings], K2 serotype [green rings], non‐K1/K2 serotype [gray rings]), and cKp (blue rings) isolates. Annotated regions indicate the genes encoding the virulence factors rmpA, rmpA2, aerobactin (iucABCD‐iutA), and salmochelin (iroBCDN)

**FIGURE 3:**
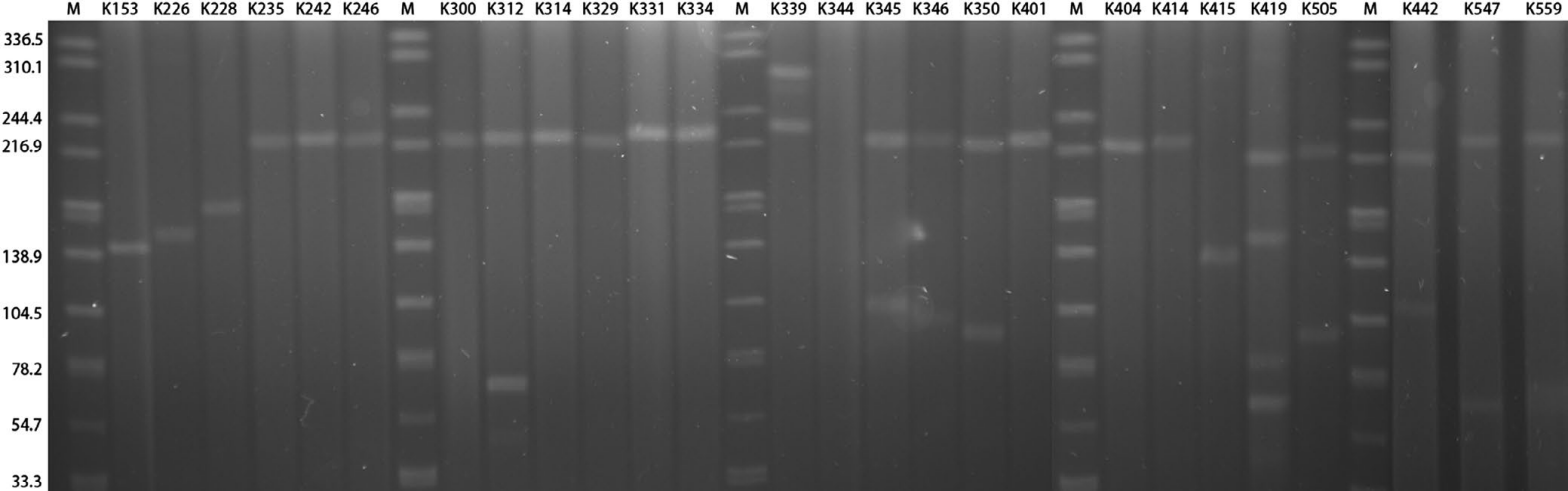
S1‐PFGE results of hvKp isolates

Among the 26 hvKp isolates, 24 strains harbored a virulence plasmid with a high homology to pNTUH‐K2044 and included 13 K1 serotype, 7 K2 serotype, 3 K64 serotype, and 1 K54 serotype strains (Figure 2). Moreover, the S1‐PFGE and BLASTN atlas illustrated that all the 13 K1 serotype strains contained a virulence plasmid with a size similar to that of pNTUH‐K2044 (around 220 kbp), while 7 K2 serotype strains harbored a virulence plasmid with different sizes, ranging from 140 to 220 kbp. Interestingly, 6 strains harbored another 1‐3 plasmids, besides a virulence plasmid (Figure 3). One K5 serotype and one K64 serotype strain did not exhibit a virulence plasmid. Both contained the ICEkp1 virulence island in its chromosome. The detection rates of virulence genes for individuals infected with hvKp, including colibactin, aerobactin, salmochelin, rmpA, and rmpA2, were higher than those for individuals infected with cKp (Table 3).

**TABLE 3:** Virulence characteristics of 26 hvKp and 25 cKp isolates

Virulence factors	No. of isolates (n, %)		*P* value
	hvKp (n = 26)	cKp (n = 25)	
String test	23 (88.5)	0 (0.0)	
Aerobactin	25 (96.2)	1 (4.0)	
RmpA	24 (92.3)	0 (0.0)	
RmpA2	23 (88.5)	0 (0.0)	
Serotype			
K1	13 (50.0)	0 (0.0)	
K2	7 (26.9)	3 (12.0)	
K5	1 (3.9)	0 (0.0)	
K54	1 (3.9)	1 (4.0)	
K64	4 (15.4)	0 (0.0)	
Other	0 (0.0)	21 (84.0)	
Yersiniabactin	18 (69.2)	9 (36.0)	.017
Colibactin	13 (50.0)	0 (0.0)	.000
Salmochelin	26 (100.0)	0 (0.0)	.000
ICEKp	18 (69.2)	8 (32.0)	.008
ICEKp1	2 (7.7)	0 (0.0)	
ICEKp3	3 (11.5)	2 (8.0)	
ICEKp4	0 (0.0)	2 (8.0)	
ICEKp5	0 (0.0)	1 (4.0)	
ICEKp10	13 (50.0)	0 (0.0)	
ICEKp11	0 (0.0)	3 (12.0)	
Virulence plasmid	24 (92.3)	0 (0.0)	.000

## 4 DISCUSSION

As a new variant of *K. pneumoniae*, hvKp could cause life‐threatening and metastatic invasive infections. The poor outcome might be attributable to a delayed diagnosis and lack of scheduled antibiotic treatment.^2^ Unfortunately, the definition of hvKp remains controversial. Hypermucoviscosity was considered an important in vitro parameter for the identification of hvKp,^3^, ^4^ though some reports indicated an inconsistency in the association between the hypermucoviscosity phenotype and virulence.^17^, ^18^ Recently, studies have increasingly defined hvKp based on the genetic background, involving genes such as aerobactin (iucABCD‐iutA), salmochelin (iroBCDN), and regulators of the mucoid phenotype (rmpA and rmpA2).^9^, ^12^, ^19^, ^20^ Here, we defined hvKp based on both genetic background and hypermucoviscosity phenotype. Notably, 4 hvKp strains with the nonhypermucoviscosity phenotype were identified, of which 2 were KPC2‐producing strains. The BLASTN atlas showed that these 2 carbapenem‐resistant hvKp strains harbored the virulence plasmid, which exhibited a high homology to pNTUH‐K2044, along with the loss of the rmpA gene, resulting in the nonhypermucoviscosity phenotype. Therefore, it might not be appropriate to define hvKp based on the hypermucoviscosity phenotype alone.

Since it was first described in Taiwan in 1986, hvKp infections have spread worldwide. Initially, most of the infections caused by hvKp were reported to be sporadic community‐acquired infections. Recently, reports of nosocomial infections caused by hvKp, and especially ESBL‐producing and carbapenem‐resistant hvKP, have increased.^12^, ^19^ The drug resistance phenotype of hvKp could occur as a result of either acquisition of resistance plasmids by hvKp strains or horizontal transfer of a virulence plasmid with high homology to pNTUH‐K2044 into multi‐drug resistant strains.^11^, ^21^, ^22^, ^23^, ^24^ The spread of both hvKp and carbapenem‐resistant *K. pneumoniae* infections occurred as epidemic in China.^9^, ^25^ The hospital environment could be regarded as a reservoir for antibiotic resistance genes and play a role in the outbreak of multi‐drug resistant bacteria.^26^, ^27^ It was likely that the high prevalence of hvKp and carbapenem‐resistant *K. pneumoniae* in Chinese hospitals contributed to the emergence of carbapenem‐resistant hvKp organisms.^28^ Several reports of sporadic cases or small outbreaks mostly associated with nosocomial infections emerged due to the presence of carbapenem‐resistant hvKp strains in China.^11^, ^22^, ^23^, ^24^, ^29^, ^30^, ^31^ In this study, we isolated 26 hvKp strains (26/51, 51.0%) from wound drainage specimens obtained from postoperative patients. According to previous reports, hvKp strains were rarely resistant to any of the commonly used antimicrobial agents, with the exception of ampicillin.^32^ However, in this study, 4 ESBL‐producing strains (4/26, 15.4%) and 2 carbapenem‐resistant strains (2/26, 7.7%) were found. There were no significant differences in the incidence of secondary surgery, duration of hospitalization, and 30‐day mortalities between the hvKp and cKp groups. In comparison to patients with cKp infections, patients with hvKp infections displayed higher rates of multi‐organ failure, but the mortality rates were not significantly different.^33^ However, the 14‐day cumulative survival periods were significantly different between the blaKPC(+) and blaKPC(‐) subgroups,^13^ suggesting that resistance had a notably greater impact on hvKp infection prognosis than virulence. The emergence of ESBL‐producing and carbapenem‐resistant hvKp strains indicated the importance of epidemiologic surveillance and clinical awareness of this pathogen, especially in clinical settings.^20^, ^34^

K1 and, to a lesser extent, K2 were the major serotypes of hvKp strains. Among the 26 hvKp isolates, 13 K1 serotype (13/26, 50.0%) and 7 K2 serotype (7/26, 26.9%) strains were identified, respectively. PFGE analysis showed that 13 K1 serotype isolates exhibited a high degree of clonality, while 7 K2 serotype strains were genetically unrelated. MLST analysis indicated a strong correlation between ST23 and the K1 serotype. ST65, ST86, and ST375 were prevalent in K2 serotype strains. Despite their different genetic backgrounds, almost all of the hvKp strains (24/26, 92.3%) harbored a large virulence plasmid with a high homology to pNTUH‐K2044. This plasmid encodes two siderophores, aerobactin and salmochelin, along with rmpA (regulator of the mucoid phenotype); these were found to be restricted to hvKp isolates.^6^ In total, 30 genes were reported to be highly pyogenic liver abscess‐associated genes, of which 27 were located on the virulence plasmid.^15^ The lack of the virulence plasmid would significantly reduce the virulence of a CC23 strain,^17^, ^35^ indicating the important role of this virulence plasmid in the hypervirulence of *K. pneumoniae*. Interestingly, S1‐PFGE and the BLASTN atlas illustrated that all of the 13 K1 serotype strains contained a virulence plasmid with a size similar to that of pNTUH‐K2044 (around 220 kbp), while 7 K2 serotype strains harbored a virulence plasmid with a size ranging from 140 to 220 kbp. The virulence plasmids were found to occur in a growing number of distinct lineages, suggesting that acquisition had occurred, albeit at a very low frequency,^36^ and the insertion or loss of genetic material might have occurred during the horizontal transfer of the virulence plasmid.

In summary, we have shown that hvKp (defined as aerobactin positive) strains were prevalent in SSI infections in China. K1 and K2 were the major serotypes of hvKp, and almost all the hvKp strains (24/26, 92.3%) harbored a large virulence plasmid with a high homology to pNTUH‐K2044. Effective surveillance and control measures should be implemented to prevent the dissemination of such organisms, including the ESBL‐producing and carbapenem‐resistant hvKp strains.

## ETHICAL APPROVAL

Not applicable.
